# P-1609. Provider and Nursing Perspectives on "Panculture": Opportunities for Innovative Diagnostic Stewardship Interventions

**DOI:** 10.1093/ofid/ofae631.1776

**Published:** 2025-01-29

**Authors:** Kevin Gibas, Leonard Mermel

**Affiliations:** Rhode Island Hospital, Pawtucket, Rhode Island; Warren Alpert Medical School of Brown University, Providence, RI

## Abstract

**Background:**

Overutilization of diagnostic tests results in inappropriate antibiotic use, increased hospital length of stay, higher healthcare costs, overdiagnosis of healthcare-associated infections, and antimicrobial resistance.

Table 1

**Figure:**
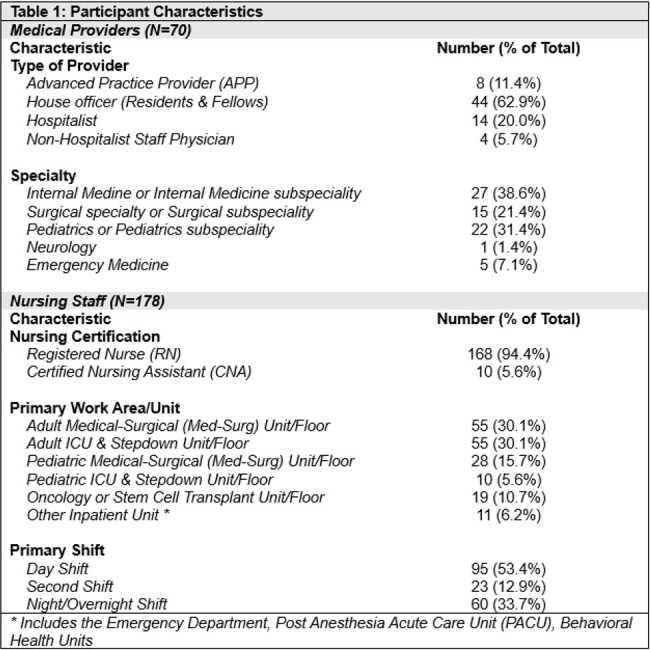

Participant Characteristics

**Methods:**

From January 3 – February 26, 2024, we conducted two prospective surveys focused on the evaluation of febrile inpatients at Rhode Island Hospital. One survey was of medical providers trained in internal medicine, surgery, pediatrics, emergency medicine, and neurology and the other was of nursing staff in inpatient areas and the emergency department (ED).

**Figure:**
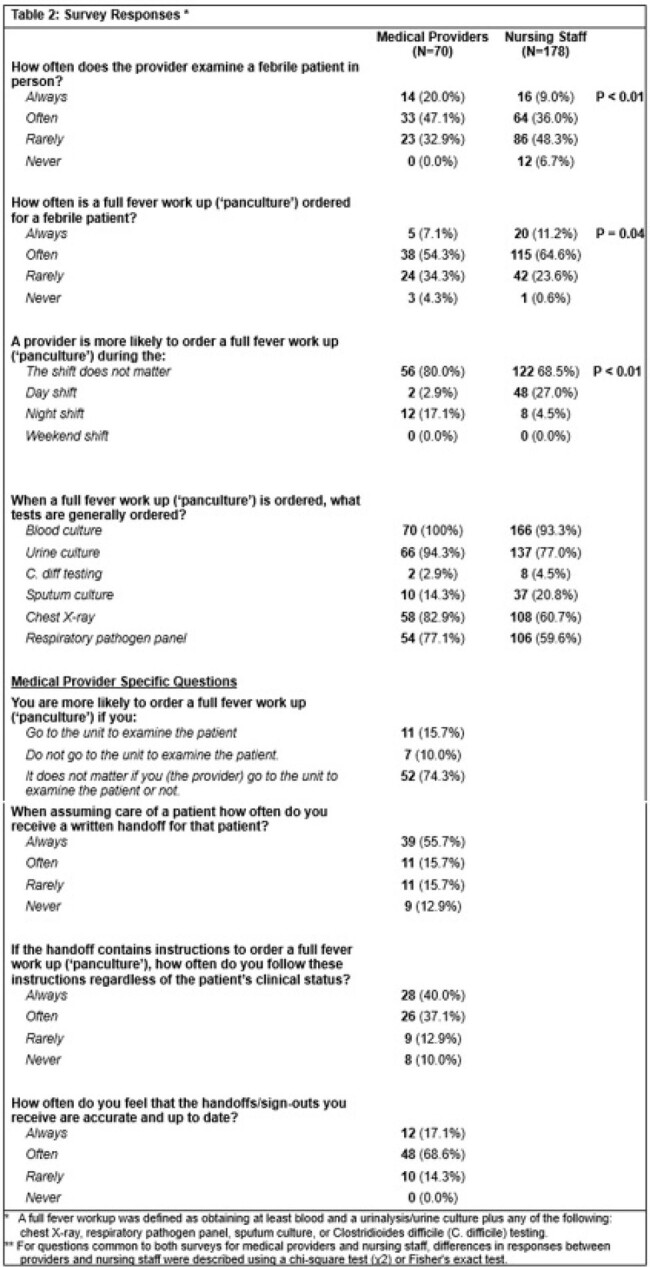

**Results:**

70 providers (9%) and 178 nursing staff (12%) completed their respective surveys. When asked about evaluating febrile inpatients, 64% of providers (n=43) reported “always” or “often” ordering full fever workups or ‘panculturing’ and 67% of providers (n=47) reported “always” or “often” physically evaluating febrile patients at the bedside (Table 2). In contrast, only 45% of nursing staff (n=80) reported that providers “always” or “often” evaluate febrile patients in person. 76% of nursing staff (n=135) responded providers “always” or “often” order full fever workups. When asked about hand-off practices for febrile patients, 71% of providers (n=50) reported “always” or “often” receiving written hand-offs. 86% of providers (n=60) reported the hand-offs are “always” or “often” accurate; however, only 17% of providers responded these were “always” accurate. 77% of providers (n=54) reported “always” or “often” following hand-off instructions to obtain a full fever workup if a patient becomes febrile, regardless of a patient’s clinical status. Responses differed significantly by nursing unit type (Table 3) and by provider specialty and position (Tables 3 & 4).

Table 3

**Figure:**
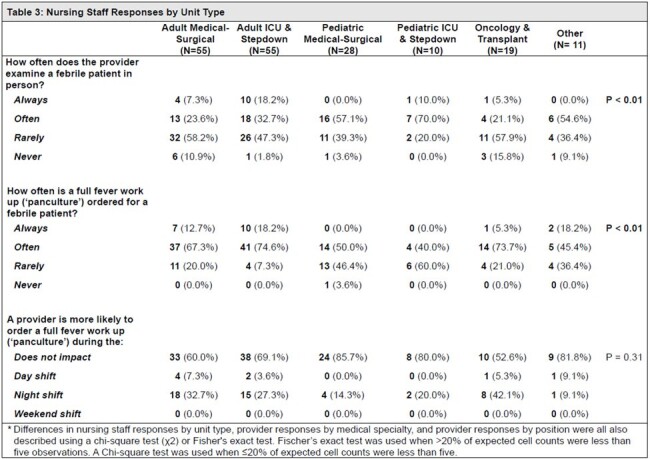

Nursing Staff Responses by Unit Type

**Conclusion:**

This study elucidates drivers of inefficient and excessive utilization of diagnostic studies at RIH and identifies potential targets for diagnostic stewardship interventions.

Table 4

**Figure:**
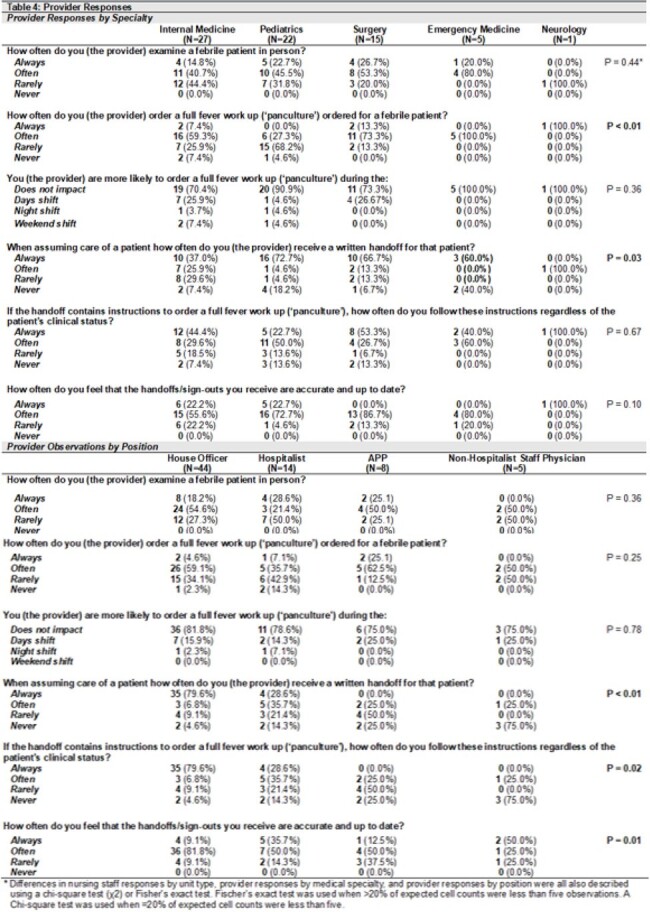

Provider Responses by Specialty and Position

**Disclosures:**

**Leonard Mermel, DO, ScM**, Citius Pharmaceuticals: Advisor/Consultant|CorMedix Pharma: Advisor/Consultant|Destiny Pharma: Advisor/Consultant|Lightline Medical: Advisor/Consultant|Lightline Medical: Stocks/Bonds (Private Company)|Pristine Access Technologies: Advisor/Consultant|Pristine Access Technologies: Stocks/Bonds (Private Company)

